# The Development of Executive Function in Autism

**DOI:** 10.1155/2012/146132

**Published:** 2012-07-05

**Authors:** Elizabeth Pellicano

**Affiliations:** ^1^Centre for Research in Autism and Education (CRAE), Department of Psychology and Human Development, Institute of Education, London WC1H 0AA, UK; ^2^School of Psychology, University of Western Australia, Crawley, WA 6009, Australia

## Abstract

Autism is a common and often highly debilitating neurodevelopmental condition, whose core behavioral features are believed to be rooted in disrupted neurocognitive processes, including especially “executive function.” Researchers have predominantly focused upon understanding the putative causal relationship between difficulties in EF and autistic symptomatology. This paper suggests, however, that the effects of individual differences in EF should be more far-reaching, playing a significant part in the real-life outcomes of individuals with autism, including their social competence, everyday adaptive behavior, and academic achievement. It further considers the nature of the EF-outcome relationship, including the possible determinants of individual differences in EF, and makes several recommendations for future research.

## 1. Introduction

Autism spectrum conditions (hereafter, “autism”) are a set of common, lifelong neurodevelopmental conditions that involve substantial heterogeneity at numerous levels, including etiology, neurobiology, cognition, and especially behavior. Long-term follow-up studies show that the developmental outcomes of autistic individuals are highly variable, even for individuals at the more intellectually able end of the autism spectrum. While some individuals go on to live independently and obtain qualifications, the majority fail to achieve independence, to attain full-time employment, or to enjoy friendships [1–5] (though see [6]). Explaining this variability is of critical import: to discover why developments take place in some areas and not in others, and especially in some individuals and not in others. 

Researchers have little understanding of the factors underpinning this heterogeneity, due in part to a dearth of longitudinal studies tracing the development of autism and to the preponderance of studies using a case-control design, focusing on group rather than on individual differences. This paper aims to address this limitation. It identifies one potential source of this variability, namely, autistic children's emerging “executive function” (EF), those higher-order processes, closely associated with the prefrontal cortex, which are necessary for regulating and controlling behavior (see Table 1). Specifically, it suggests that individual differences in the growth trajectories of autistic children's EF skills could explain some of the variability in children's functional outcomes, including their social awareness, real-life adaptive behavior, and readiness to learn in school, both in the shorter term and in the longer term. 

## 2. Executive Functions in Autism

EF has received extensive attention in the autism literature for many years largely due to the influential proposal that the inherent rigidity and invariance of autistic behaviors could be explained by a primary impairment in executive control [7, 8]. EF problems have been demonstrated consistently in school-age children, adolescents, and adults with autism [8, 9], as well as relatives of individuals with autism albeit to a lesser degree [10, 11] (see [12, 13] for reviews). These problems typically manifest as perseverative responses (i.e., getting “stuck” performing the same action) and difficulties switching flexibly between response sets. Furthermore, problems in specific components of EF have been shown to distinguish autism from other developmental conditions, such as attention deficit/hyperactivity disorder (ADHD) [14, 15] (though see [16]). 

Despite the theory's apparent face validity, several negative findings have cast doubt over the possibility that a *primary *problem in EF can explain all autistic symptoms. Not all individuals show EF difficulties (e.g., [17–19]), and investigations of EF in young children with autism have failed to find evidence of autism-specific difficulties [20, 21]. Consequently, researchers have shifted away from a framework that emphasizes a single, primary neurocognitive atypicality (such as EF) as the underlying cause of autism to one that encompasses multiple cognitive atypicalities [22, 23]. 

## 3. Why Focus on Autistic Children's Developing Executive Function?

While there is general consensus that EF problems are unlikely to play a primary causal role in autism, it remains possible that the degree of difficulties in EF could play a substantial role in autistic children's developmental outcomes—including their social competence (those skills, including “theory of mind,” which allow individuals to evaluate social situations and respond effectively; see Table 1), their adaptive behavior (those skills necessary for individuals to live independently and to function well in real-life settings), and their success in school. Indeed, clinicians and those who care for individuals with autism often associate some individuals' inability to achieve independence with persistent difficulties in regulating behavior and adapting flexibly to change (e.g., [2]). Therefore, whether poor EF plays a fundamental role in the emergence of core autistic features or, instead, is a consequence of early atypical input from another cognitive system, it is nevertheless likely to place the child with autism at risk for a poor developmental outcome either directly or indirectly. 

Substantial recent progress in understanding the typical development of EF provides further reason to investigate individual differences in EF as a possible source of the heterogeneity in outcomes in autism. First, it is clear that EF is intimately tied to another aspect of neurocognitive development known to develop atypically in autism: theory of mind (ToM: mental state awareness). Numerous studies report robust correlations between individual differences in tasks tapping ToM and EF, independent of the effects of age and IQ, in typical preschoolers [24–27] and toddlers [28, 29]. Several theorists have further proposed a *direct*, functional link between EF and ToM. They assert that the abilities to monitor one's actions and to act with volition are critical for reflecting on the mental states of self and other, that is, that ToM emerges from EF [30, 31]. Research findings have repeatedly shown that early EF skills are predictive of later ToM but early ToM skills are not predictive of later EF [27–29], providing overwhelming support for the view that EF is critical to developmental changes in ToM.

Second, early EF is also predictive of typical preschoolers' later school readiness [32, 33] and academic success in reading and mathematics [34–36]. The transition to school relies on mastery of basic EF skills, including remembering and following instructions, completing tasks independently and smoothly transitioning between tasks, and inhibiting inappropriate behaviors. EF, therefore, is held to play an important role in the acquisition of knowledge; the better children are at focusing and refocusing their attention, holding information in mind and manipulating it and resisting distraction, the better placed children should be to acquire knowledge and skills in the classroom.

Third, it is well known that the prefrontal cortex, which mediates EF, shows a protracted development trajectory: it begins to develop very early in life, has a boost during the preschool period, and continues to develop well into adolescence [37]. The extended postnatal developmental of prefrontal cortical networks means that they should be particularly sensitive to exogenous influences. Several studies now report direct evidence of the malleability of EF: “exercising” young children's EF skills results in substantial improvements in their ability to regulate their behavior in both at-risk [38] and typical [39] populations.

Taken together, these findings provide compelling grounds for suggesting that one source of the heterogeneity in autistic individual's functional outcomes are individual differences in emerging EF. Some researchers have begun to speak to this possibility, with several studies reporting links between EF and a range of concurrent behaviors, including social competence (e.g., [18, 40]) and everyday adaptive behavior (e.g., [41, 42]). Yet full consideration of this *developmental* issue has not yet been possible due to a paucity of studies analyzing individual differences within a longitudinal design. The handful of studies using such an approach have done so, though, with dramatic success. Griffith et al. [20] found links between early executive skills (performance on a spatial reversal task tapping cognitive flexibility) and sociocommunicative behavior (bids for joint attention) one year later in young children with autism, but not in nonautistic children with developmental delay. Also, individual differences in set-shifting ability predicted the social competence scores of cognitively able adults with autism 3 years later in one study [43] and, in another, predicted real-life adaptive skills between 11 and 27 years later [5].

A recent study has further demonstrated the influence of autistic children's early EF skills on their sociocognitive awareness. Pellicano [18] investigated the EF and ToM skills of 37 cognitively able children with autism and 31 typical children (M age = 5 yrs 6 mths). As expected, children with autism showed difficulties in both domains compared to typical children. There were also significant EF-ToM correlations, suggestive of a functional link between domains. Analysis of patterns of “atypicalities,” however, revealed striking dissociations in one direction only: poor ToM coupled with intact EF [18]. In line with Russell [31], these findings suggested that EF skills might be one important ingredient for the development of ToM but that ToM does not play this role for the development of EF.

Three-year follow-up data on the same samples of children further supported this claim [54]. For children with autism, individual differences in early EF skills (Time 1) were longitudinally predictive of developmental change in ToM skills (Time 2), independent of age, language, nonverbal intelligence, and early ToM skills. Yet there were no predictive relations in the opposite direction—a finding that is entirely consistent with longitudinal studies of the EF-ToM relation in typical children [27–29]. These results provide initial evidence that autistic children's cognitive skills emerge within a dynamic developing system where EF skills play a critical role in shaping the developmental trajectories of ToM. 

In sum, accumulating evidence of the important contribution of EF in typical development, together with promising findings from studies with individuals with autism, provides good reason to suspect that individual differences in the development of EF might critically influence autistic children's developmental trajectories and could account, at least in part, for the heterogeneity in their sociocognitive, behavioural and academic success.

## 4. How Is EF Organized in Autism and Which Factors Drive Its Growth? 

Determining the precise nature of the developmental course of EF in autism and also of the potential causal links between EF and other social and learning outcomes demands greater understanding of the nature of EF itself and of the mechanisms underpinning its growth. Although there is no question that EF plays a vital role in well-regulated, organized behavior [55], there has been much disagreement regarding the characterization of the EF construct. Like adult models [56, 57], competing developmental accounts differ with respect to which they emphasize the unitary [58, 59] or fractionated [60, 61] nature of EF. Empirical work with adults, using sophisticated statistical techniques like confirmatory factor analysis (CFA), has reported evidence of three latent EF variables or component processes—set-shifting, updating (working memory), and inhibitory control—which are partially independent but still intercorrelated [62]. 

**Figure 1:**
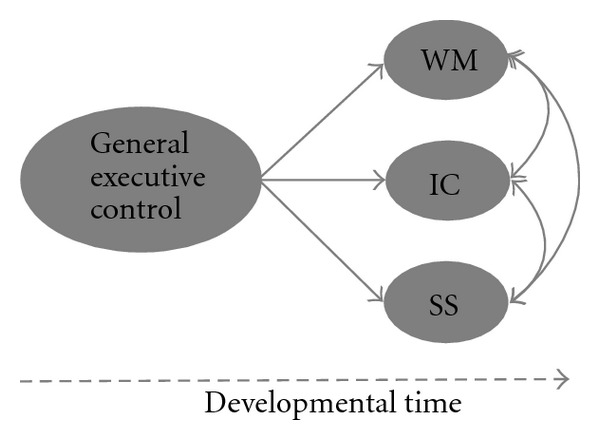
A simplified model showing a unitary executive function (EF) construct early in development and an emergent fractionated construct of EF with development with latent EF variables of working memory (WM), inhibitory control (IC), and set-shifting (SS).

Studies using similar methods with school-age children in part support this integrated framework [63, 64], although recent studies with 2- to 6-year-old children have instead reported evidence in line with a unitary model of EF [65, 66]. These latter results question the apparent continuity in the structure of EF during development but are consistent with a dynamic, neuroconstructivist approach in which cognitive functions begin relatively undifferentiated and become progressively modularised or specialised over time [67] (see Figure 1). This framework suggests that individual differences in development itself might be key to explaining the wide variation in findings both within [68] and across [12, 16] studies on EF in autism. 

No study has yet explicitly investigated the nature of EF in autism. Early fractionation of EF makes it plausible for distinct EF components, such as cognitive flexibility, to be specifically affected in autism. Yet if, as the evidence suggests, EF is a single, unitary construct during early childhood (at least in typical children), then it becomes more difficult to see how a distinct profile of EF difficulties might emerge in autism. Given the prolonged development of EF and the degree of neural plasticity during childhood [69, 70], it is likely that emerging prefrontal cortical networks affect, and are affected by, the development of other key cognitive functions. In this case, then, disruption to distinct EF components in autism might be driven by other factors.

**Figure 2:**
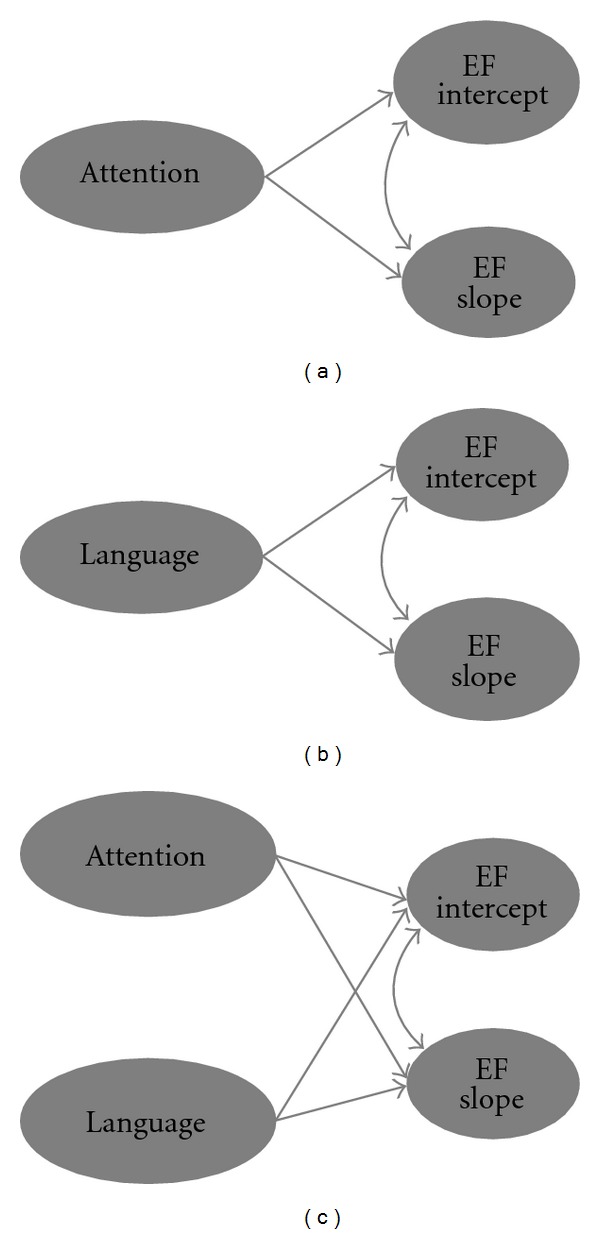
Three simplified models showing executive function (EF) development. Model (a) includes a latent construct of attention that influences baseline levels (EF intercept) and rate of growth of EF (EF slope). Model (b) shows a latent construct of language mediating baseline EF and its developmental trajectory. Model (c) includes latent constructs of attention and language, which both influence the development of EF. These models could be tested using latent growth curve modelling.

One goal therefore should be to understand precisely which mechanism(s) *drives* the development of EF in typical children and in children with autism. Some theorists propose that progress in EF occurs via the development of the prefrontal cortex [60] and the strengthening of prefrontal representations [58] in an experience-dependent manner [71]. An influential yet contrasting view suggests that development in children's *attentional control*—the ability to focus on a task and ignore irrelevant information—is the source of common variance in EF [61, 72]. Posner et al. have demonstrated significant advances during the preschool period in the central “attention network,” which includes alerting, orienting, and executive attention processes [73, 74]. Developmental changes in attention are considered to provide children with greater executive control over action. On this view, then, rate of growth in EF should be predicted by developments in attentional capacities (Figure 2(a)) and such developments might even mediate the fractionation of EF. 

Impairments in core attentional processes have been reported in autism, including problems with disengagement or so-called “sticky” attention [75–77]. Fundamental problems in critical aspects of attention could therefore place limits on the rate of EF development, which could in turn hinder the emergence of autistic children's social and learning outcomes. It is of course possible that the causal relationship might exist in the opposite direction, such that early developments in EF might influence the emergence of attentional networks. The relationship between aspects of attention and components of executive control has, however, been hitherto unaddressed in autism. 

Yet Posner and Rothbart's [72] model (see also [61]) neglects the potentially mediating role of another key function: *language*. Children's verbal skills can affect the expression of EF, for example, by limiting their ability to store phonological information in working memory [78]. Yet language might play a more fundamental role, affecting the very *development* of EF. For several theorists [31, 79–81], language provides an internal plan for behavior. Vygotsky [81] emphasized the importance of self-directed speech during early childhood, which becomes increasingly internalized during the preschool years, allowing children (verbally) to “think through” problems and to guide future-oriented behavior. Similarly, Zelazo et al. [59] stress that language is the medium through which higher-order (if-if-then) rules are formulated and is key to recursive thought. Developmental gains in young children's language skills (specifically their ability to formulate hierarchical rules) are therefore directly implicated in the rate of EF development (Figure 2(b)). 

Difficulties with communication are a core characteristic of autism [82] and has been previously implicated as a potential limiting factor on the development of EF (e.g., [83]). Furthermore, children with autism are less likely to use verbal rehearsal strategies on executive tasks [84–86] suggesting that they, unlike typical children, may not be using internal language in the service of executive control. Pellicano's [54] longitudinal work showed that autistic children's initial receptive-vocabulary skills were not predictive of EF performance three years later, suggesting further that verbal skills may not influence the emergence of EF in autism as they do in typical development [26, 28]. Individual differences in the growth trajectories of autistic children's verbal skills therefore might partially mediate (or fail to mediate at all) the rate of progress of EF in autism. Further still, it is possible that attention *and* language could mediate the development of EF. In this case, both functions could make independent contributions to the rate of growth of EF (Figure 2(c)) and both could be limiting factors in autism. Importantly, evidence for any one of these patterns (Figures 2(a)–2(c)) would suggest that the potential influence of EF on children's functional outcomes is *indirect* rather than direct. 

All of these models suggest that the development of EF itself might be shaped by certain endogenous factors, which in turn could mediate children's developmental outcomes. Yet the developmental trajectory of EF, and its resulting neurocognitive architecture, will be an emergent property of interactions within the children and between the children and their environment. Alternative explanations therefore place the development of EF squarely in the social realm. For example, Luria emphasized that “we must go beyond the limits of the individual organism and examine how volitional processes are formed for the child in his/her concrete contacts with adults” [87, page 89] (see also [88]). Hughes [27] (see also [80]) extended this view to suggest that the effect of EF upon the development of ToM should be indirectly influenced by the child's social environment. Since negotiating social interactions requires children to regulate their own behaviors (e.g., turn-taking, following rules in games), peer relations are likely to have positive effects on children's developing executive skills, which in turn will foster their developing ToM.

There has been renewed interest in the sociocultural predictors of EF development, which so far include socioeconomic status [89], parent scaffolding [90, 91], and parent-child interactions [92]. These exogenous factors are also likely to influence the development of EF in children with autism. Contrary to popular opinion, children with autism do not grow up in a social vacuum. Rather, they can show attachment security with caregivers [93], can engage in positive and collaborative interactions with siblings (e.g., [94]), and actively seek out their nonautistic peers [4]. It is therefore plausible that social contact could influence autistic children's developing EF, which in turn might exert its effects on key real-life outcomes. 

## 5. Future Perspectives

There is much awareness of the huge variability in autistic children's developmental outcomes but there is very little empirical knowledge of the underlying causes of this variability. Early individual differences in EF represent one candidate source of this heterogeneity. To address this issue, however, we need a richer understanding of the causal determinants of EF growth in autism, which will require prospective longitudinal studies and carefully designed training studies. Well-designed cognitive training studies will need to disentangle whether any gains are genuinely “raising the ceiling” of performance or just “speeding up development” to reach the child's given ability. Such knowledge will lead ultimately to a more nuanced theoretical, and distinctly developmental, perspective of EF in autism. Elucidating whether EF has direct or indirect longitudinal effects on children's functional outcomes is vital for knowing whether to directly “exercise” autistic children's EF skills or to concentrate instead on bootstrapping those factors (attention/language/social environment) through which EF influences children's outcomes.

## Figures and Tables

**Table 1:** Definition of key constructs and examples of tasks used to test these skills in preschool and primary school-age children.

Construct	Component	Definition	Description of an example task
	Working memory	The ability to hold information “on-line” and manipulate it. Working memory is differentiated according to whether the information is verbal or spatial in nature.	Verbal working memory can be tested using the *Digit Span* task from the Wechsler Intelligence Scales [44] in which participants are asked to repeat number sets, which progressively increase in complexity, back to the experimenter either in the same (forwards) or reverse (backwards) order. Spatial working memory can be tested using a *Spatial Span* or *Corsi Blocks *task [45] in which the child is shown a block board containing 9 cubes in fixed positions. The experimenter taps the wooden blocks (1–9) in a sequence and child is asked to repeat the sequence in the same (forwards) or reverse order (backwards). Backward span for both tasks requires manipulating the to-be-remembered elements and thus targets working memory.
	Inhibitory control	The capacity to hold a rule in mind, responding according to this rule, and resist a prepotent response.	In *Luria's hand-game* [46, 47], a test of motor inhibition, the child first imitates the experimenter's hand movements (make a fist or point a finger). Next, the child must execute the *opposite* action (i.e., when the experimenter makes a fist, the child points finger, and vice versa). Success on this task demands that the child both hold in mind an arbitrary rule and inhibit the prepotent tendency to copy the experimenter's gesture.
Executive function	Set-shifting	The ability to shift flexibly one's attentional focus.	*Card-sorting tasks* require children to switch cognitive set in response to verbal feedback. The child is shown a set of cards and must sort the cards according to one dimension (e.g., shape). The rule then changes and the child must shift to sort according to new dimension (e.g., colour). In some tasks (e.g., in the Dimensional Change Card Sort task [48]), children are explicitly told the rule change, while in others (e.g., in the teddy bear set-shifting task [26]), the rule change is implicit. Children who can switch flexibly between cognitive sets make fewer “post-switch” errors.
	Planning	The ability to formulate a plan (including selecting appropriate goals and sub-goals) and executive this plan effectively.	In the *Tower of London task *[26, 49], children are shown a peg-board containing three vertical pegs of increasing size and three beads (red, white, black) arranged in a particular configuration (“start state”). Children are then given a picture showing the beads in a different configuration (“goal state”) and instructed to move the beads from the start state to the goal state within the minimum number of moves possible while observing various rules. Success on this task requires children to plan ahead and to generate and maintain a series of moves.

	Theory of mind	The ability to infer the mental states (e.g., beliefs, desires) of others in order to make predictions about their behavior.	In the now-classic false-belief paradigm [50, 51], children watch one character (Sally) place an object (e.g., a ball) in one location (a basket) and leave the room. While the main character is absent, another character (Ann) surreptitiously moves the object from one location to another. Children are then asked to predict the main character's behavior (“Where will Sally look for her marble?”). Successful performance on such a task involves children predicting an action based on an attributed false belief.
Social competence	Joint attention	Requires the triadic coordination or sharing of attention with another person around an object or event.	Semi-structured observational schedules like the *Early Social Communication Scales* [52] are designed to elicit joint attention behaviours, including children's ability to (a) respond to the experimenter's eye gaze or gestures (e.g., pointing) to share a common point of reference and (b) initiate joint attention using eye contact and gestures to direct the experimenter's attention to the point of interest.

Adaptive behavior		Those skills necessary for individuals to live independently and to function well in real-life personal and social settings.	The *Vineland Adaptive Behavior Scales*—*Second Edition (Vineland-II)* [53] is one standardized parent-report measure designed to assess a variety of typical developmental milestones with respect to social and communicative competence and real-life daily living skills.
